# Targeted metabolomics analysis of nucleosides and the identification of biomarkers for colorectal adenomas and colorectal cancer

**DOI:** 10.3389/fmolb.2023.1163089

**Published:** 2023-06-27

**Authors:** Weifang Zheng, Mingwei Wang, Xiaoyin Chai, Fuzhen Pan, Meihui Xu, Yingchen Wang, Liuhao Lan, Feiran Hu, Zhe Zhang, Zhu Chen

**Affiliations:** ^1^ Lanxi Hospital of Traditional Chinese Medicine, Jinhua, China; ^2^ College of Chemical Engineering, Zhejiang University of Technology, Hangzhou, China; ^3^ Lanxi Red Cross Hospital, Jinhua, China

**Keywords:** colorectal cancer, colorectal adenomas, LC-MS/MS, nucleosides, serum, biomarker

## Abstract

The morbidity and mortality of colorectal cancer (CRC) have been increasing in recent years, and early detection of CRC can improve the survival rate of patients. RNA methylation plays crucial roles in many biological processes and has been implicated in the initiation of various diseases, including cancer. Serum contains a variety of biomolecules and is an important clinical sample for biomarker discovery. In this study, we developed a targeted metabolomics method for the quantitative analysis of nucleosides in human serum samples by using liquid chromatography with tandem mass spectrometry (LC-MS/MS). We successfully quantified the concentrations of nucleosides in serum samples from 51 healthy controls, 37 patients with colorectal adenomas, and 55 patients with CRC. The results showed that the concentrations of *N* ^6^-methyladenosine (m^6^A), *N* ^1^-methyladenosine (m^1^A), and 3-methyluridine (m^3^U) were increased in patients with CRC, whereas the concentrations of *N* ^2^-methylguanosine (m^2^G), 2′-O-methyluridine (U_m_), and 2′-O-methylguanosine (G_m_) were decreased in patients with CRC, compared with the healthy controls and patients with colorectal adenomas. Moreover, the levels of 2′-O-methyluridine and 2′-O-methylguanosine were lower in patients with colorectal adenomas than those in healthy controls. Interestingly, the levels of U_m_ and G_m_ gradually decreased in the following order: healthy controls to colorectal adenoma patients to CRC patients. These results revealed that the aberrations of these nucleosides were tightly correlated to colorectal adenomas and CRC. In addition, the present work will stimulate future investigations about the regulatory roles of these nucleosides in the initiation and development of CRC.

## 1 Introduction

The morbidity and mortality rate of colorectal cancer (CRC) have been increasing worldwide in recent years. CRC is the second leading cause of cancer-related mortality worldwide, posing a serious threat to human health and causing a huge social burden (Bray et al., 2018; Hyuna et al., 2021). By the early detection of colorectal adenomas and colorectal cancer through screening, the morbidity and mortality of CRC can be successfully reduced (Shaukat et al., 2021). Screening techniques for CRC and colorectal adenomas include the following four types: 1) fecal-based examination, 2) imaging examination, 3) endoscopy, and 4) liquid biopsy. Nevertheless, due to the invasive nature of the procedures and poor patient compliance, large-scale screening is difficult to perform (Jaaks et al., 2022; Zhou et al., 2022).

Nucleic acid modification is defined as the chemical modification of nucleobases and the ribose of DNA and RNA. Most often, these alterations do not alter DNA or RNA sequences, but they do affect gene expression and several physiological processes (Roundtree et al., 2017; Deng et al., 2022). To date, more than 170 RNA modifications have been identified, each of which plays a crucial role in numerous life processes. RNA methylation modification plays an essential regulatory role in numerous biological processes and is implicated in the incidence and progression of various diseases, including cancer (Ontiveros et al., 2019; Isaia and Tony, 2020). The most abundant internal modification in mRNA is *N* ^6^-methyladenosine (m^6^A) (Liu et al., 2020), which was discovered in RNA as early as the 1970s (Dubin and Taylor, 1975; Perry et al., 1975). As a typical product of RNA methylation, with the discovery of m^6^A-associated methyltransferases (e.g., METTL3), demethyltransferases (e.g., FTO), and reader proteins (e.g., YTHDC1) (Liu et al., 2014; Zhang et al., 2015; Yang et al., 2020), there is growing evidence that the m^6^A level is dynamic *in vivo* and regulates RNA splicing, RNA stabilization, and translation (Shi et al., 2020; Sung and Yoon, 2020). Moreover, aberrant m^6^A levels are tightly associated with the onset and progression of various types of cancers (Wang et al., 2020; Jiang et al., 2021).

There are four types of nucleosides in RNA, namely, adenosine (A), guanosine (G), cytidine (C), and uridine (U). Methylated nucleosides can be produced when nitrogen or carbon atoms in the nucleobase are modified by methylation. Moreover, hydrogen in the 2′-hydroxyl group in a ribose substituted by the methyl group produced 2′-O-methylated nucleosides. According to a recent study, alterations of the levels of *N* ^1^-methyladenosine (m^1^A) and m^6^A in human serum and urine are associated with increased risks of colorectal and gastric cancer (Guo et al., 2021; Hu et al., 2021). Additionally, decreased concentrations of *N* ^1^-methylguanosine (m^1^G), 2′-O-methylcytidine (C_m_), and 2′-O-methyluridine (U_m_) in serum may be associated with breast cancer (Fang et al., 2021). As a result, these methylated nucleosides may be indicators for the early detection of various types of cancers.

Most commonly, serum is used in biomarker discovery, is easy to obtain, and is less traumatic (Huang et al., 2018; Shen et al., 2021). The presence of methylated nucleosides in urine and serum has previously been linked to the development of cancer in a number of studies (Djukovic et al., 2010; Guo C et al., 2018; Fang et al., 2022; Zhang et al., 2022). Liquid chromatography with tandem mass spectrometry (LC-MS/MS) has the benefit of extremely high sensitivity to meet the efficient qualitative analysis and the accurate quantitative analysis of metabolites in complex biological samples (Guo et al., 2016; Guo M et al., 2018; Zheng et al., 2020). With the LC-MS/MS method established in this study, 11 nucleosides were quantified in 37 patients with CRC, 55 patients with colorectal adenomas, and 51 healthy controls. We demonstrated the differences of these nucleosides among these groups and evaluated the potential of these nucleosides as biomarkers of colorectal adenomas and CRC.

## 2 Materials and methods

### 2.1 Chemicals and reagents

Acetonitrile of chromatographic grade was purchased from Merck KGaA (Darmstadt, Germany). A solution of formic acid (HCOOH) was purchased from Fluka (Muskegon, USA). Ammonium formate and malic acid were obtained from Sigma-Aldrich (St Louis, MO, United States). Adenosine, *N* ^6^-methyladenosine, *N* ^1^-methyladenosine, 2′-O-methylguanosine (G_m_), *N* ^1^-methylguanosine, *N* ^2^-methylguanosine (m^2^G), 2′-O-methylcytidine, uridine, 3-methyluridine (m^3^U), 5-methyluridine (m^5^U), and 2′-O-methyluridine and isotopically labeled standards, including [^13^C_5_] A, [D_3_] m^6^A, [D_3_] m^1^A, [D_3_] C_m_, [^13^CD_3_] m^5^C, [^13^C_5_N_2_] U, [D_3_] U_m_, [^13^C_5_] m^5^U, and [D_6_] m^2,2^G, were purchased from Toronto Research Chemicals (Toronto, Canada). In order to purify water, a Milli-Q water purification device was used (Millipore, Milford, MA, United States).

### 2.2 Instrumentation

The ACQUITY UPLC system (Waters, Milford, MA, United States) was used to analyze nucleosides and methylated nucleosides. For the MS analysis, an AB SCIEX 4000 QTRAP mass spectrometer (Foster City, CA, United States) was used. A Waters BEH Amide column (2.1 × 100 mm; 1.7 µm) was used for chromatographic separation. Data were acquired using the multiple-reaction monitoring (MRM) mode in the electrospray ionization (ESI) positive-ion mode. Data collection and processing were conducted using Analyst 1.6.3 software.

### 2.3 Sample collection

In this study, colorectal cancer patients, colorectal adenoma patients, and healthy controls were recruited from the Lanxi Hospital of Traditional Chinese Medicine. This study complies with the Helsinki Declaration of the World Medical Association. A total of 37 colorectal cancer patients, 55 colorectal adenoma patients, and 51 healthy controls were studied. Information about these volunteers can be found in Supplementary Table S1. Serum was collected early in the morning and was stored at −80°C until analysis.

### 2.4 Sample preparation

The serum sample of 100 μL was placed into a 1.5-mL centrifuge tube, thawed in ice, and 10 μL of the internal standard isotope (IS) was added. To thoroughly remove the protein, 330 μL of pre-cooled methanol/acetonitrile (2:1, v/v) was added, and the mixture was vortexed for 1 minute and placed at −20°C for 2 h. Extraction was performed by centrifuging the obtained mixture at 13,000 rpm at 4°C. A volume of 352 μL of the supernatant was taken and evaporated under vacuum. A volume of 80 μL of water/acetonitrile (9:1, v/v) was used to dissolve the residues. Finally, the solution was analyzed by LC-MS/MS.

### 2.5 LC-MS/MS analyses

The mobile phase A was a solution of 10 mM ammonium formate, 0.2% formic acid, and 0.05 mM malic acid in water. The mobile phase B was composed of a solution containing 2 mM ammonium formate, 0.2% formic acid, and 0.05 mM malic acid in acetonitrile. The 11 analytes were separated at a flow rate of 0.3 mL/min using gradient elution, shown as follows: 0–5.5 min, 5% A; 5.5–7 min, 5%–8% A; 7–9 min, 8%–15% A; 9–9.5 min, 15%–17% A; 9.5–11 min, 17%–20% A; 11–11.5 min, 20%–5% A; and 11.5–15 min, 5% A. Each sample was tested at an injection volume of 5 µL.

The voltage of 5.5 kV was used in the ion spray process. The ion source (TEM) was set at 550°C. Both ion source gases (GS1 and GS2) were set as 50 psi. The pressure of the curtain gas (CUR) was set at 40 psi. The declustering potential (DP), entry potential (EP), collision energy (CE), and collision cell exit potential (CXP) were optimized, as shown in Supplementary Table S2.

### 2.6 Statistical analysis

GraphPad Prism 8 was used to perform the statistical analysis. The levels of nucleosides in 51 healthy controls, 55 patients with colorectal adenomas, and 37 patients with colorectal cancer were compared using the Mann–Whitney test. The statistics were defined as significant when the *p*-value was less than 0.05.

## 3 Results and discussion

### 3.1 Establishment of the targeted metabolomics method for the analysis of nucleosides

**FIGURE 1:**
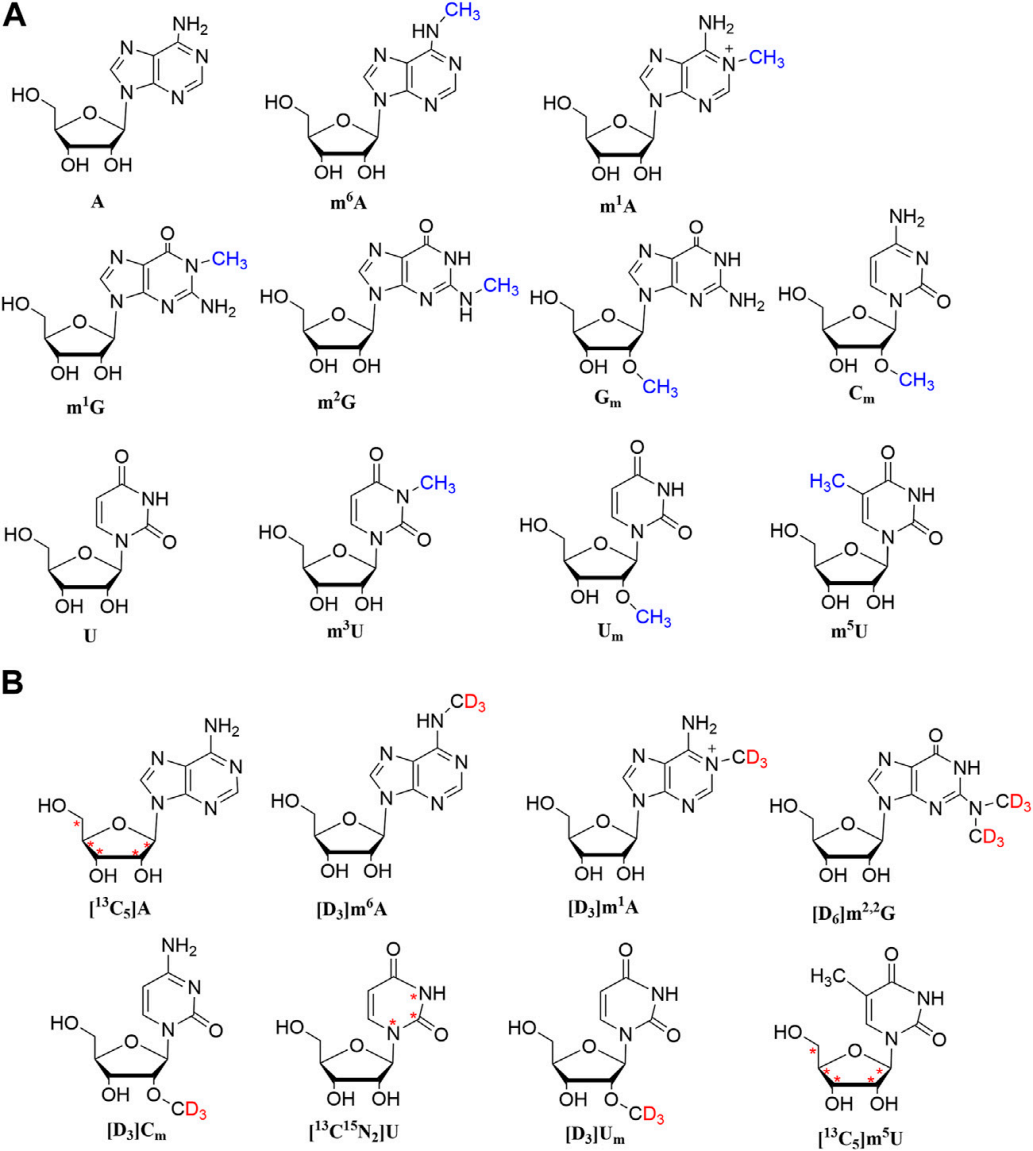
Chemical structures of A, m^1^A, m^6^A, U, m^3^U, m^5^U, U_m_, C_m_, m^2^G, m^1^G, and G_m_ and their stable isotope-labeled internal standards. Asterisk (*) designates the of ^13^C labeling.

**FIGURE 2:**
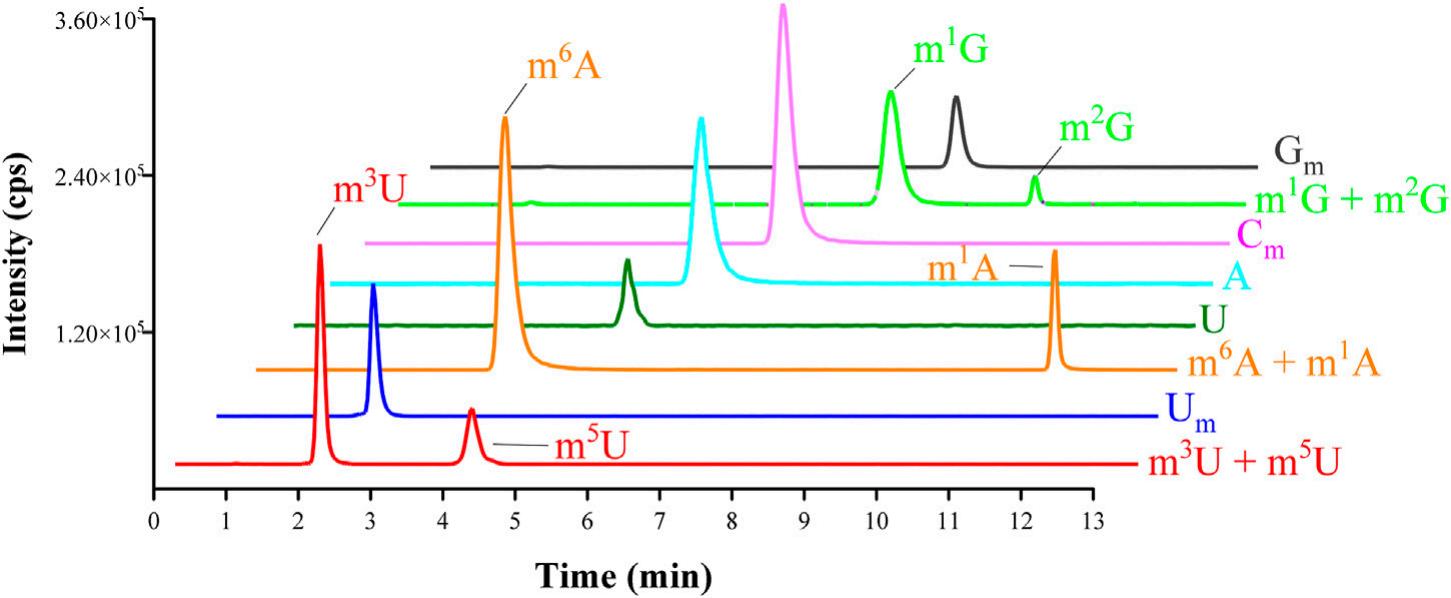
MRM chromatograms of A, m^1^A, m^6^A, U, m^3^U, m^5^U, U_m_, C_m_, m^1^G, m^2^G, and G_m_ standards.

The chemical structures of nucleosides are shown in Figure 1. In addition, a BEH Amide column (2.1 × 100 mm, 1.7 µm) was chosen. As depicted in Figure 2, the analysis was completed in 12 min. The target analytes were perfectly separated, demonstrating that the approach has high separation efficiency, fast analysis speed, and high throughput. Hence, it is suited for the analysis of a large number of clinical samples.

To optimize the MRM parameters, a direct infusion of the standard solution into the mass spectrometer by using a peristaltic pump was performed. In full-scan ESI-MS, [M + H]^+^ ions at *m/z* 268.1 were found for A. Abundant [M + H]^+^ and M^+^ ions at *m/z* 282.1 were detected in m^6^A and m^1^A, respectively. For m^1^G, m^2^G, and G_m_, abundant [M + H]^+^ ions at *m/z* 298.1 were observed. For m^3^U, m^5^U, and U_m_, abundant [M + H]^+^ ions at *m/z* 259.1 were observed. In addition, [M + H]^+^ ions at *m/z* 245.1 for U and *m/z* 258.1 for C_m_ were observed. Subsequently, a collision-induced dissociation (CID) was carried out. For A, CID data showed that the most common fragment ion was at *m/z* 136.0. For m^1^A and m^6^A, the most abundant fragment ions were both at *m/z* 150.0, and for G_m_, the most abundant fragment ion was at *m/z* 152.0. For m^1^G and m^2^G, the most abundant fragment ions were both at *m/z* 166.0. For m^3^U and m^5^U, the most abundant fragment ions were both at *m/z* 127.0. Furthermore, the most abundant fragment ions for U and C_m_ were found to be at *m/z* 112.0 and *m/z* 113.0, respectively. Then, the MRM parameters of these nucleosides and their isotope-labeled internal standards were optimized, as shown in Supplementary Table S2.

### 3.2 Targeted metabolomics analysis of nucleosides and in-serum samples from patients with colorectal adenomas, patients with CRC, and healthy controls

**FIGURE 3:**
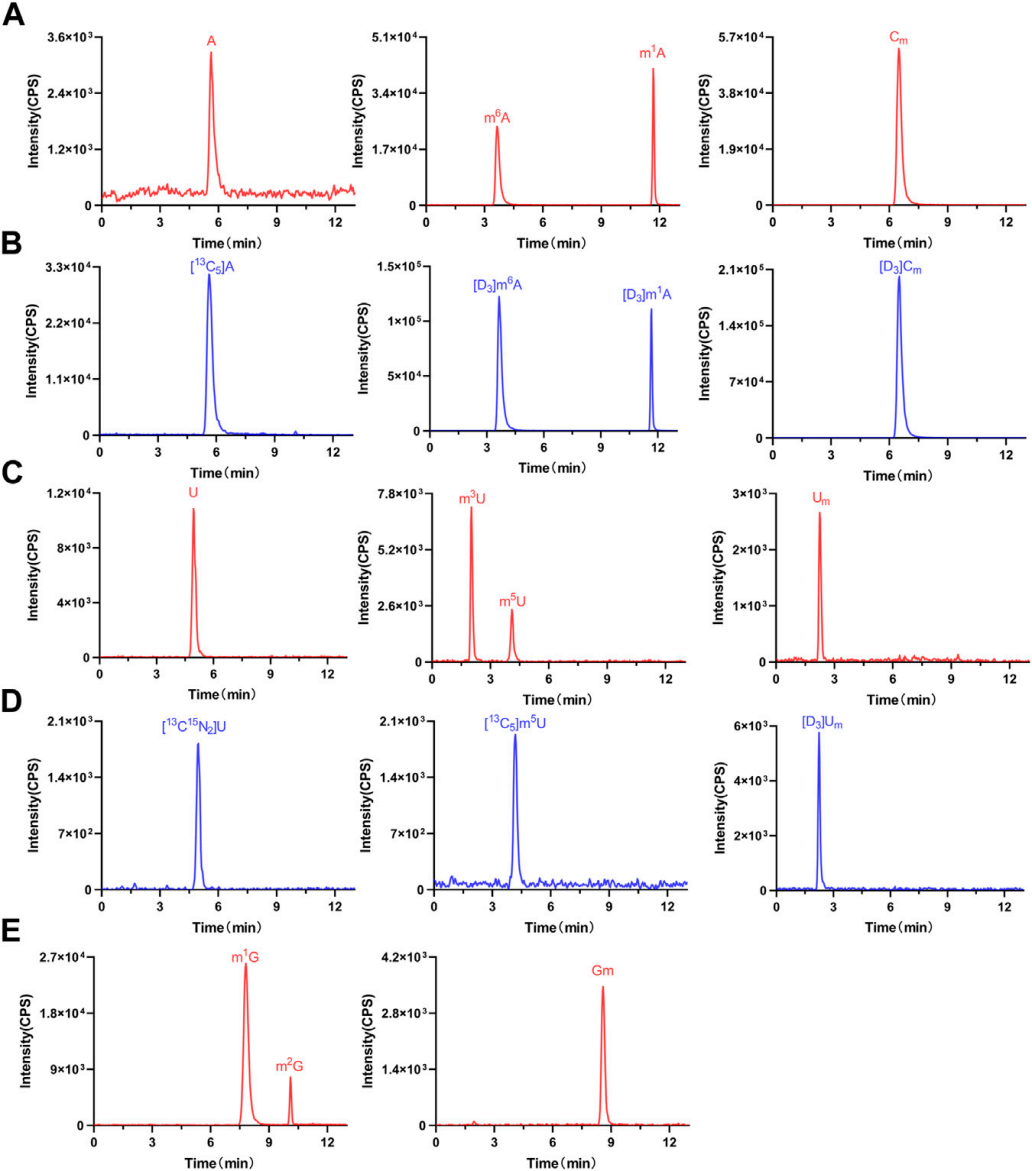
Representative MRM chromatograms of **(A)** A, m^1^A, m^6^A, C_m_, **(C)** U, m^3^U, m^5^U, U_m_, **(E)** m^1^G, m^2^G, and G_m_ and **(B) (D)** spiked isotope-labeled internal standards in a serum sample.

By establishing the LC-MS/MS method described previously, we detected these nucleosides in serum samples from 37 colorectal cancer patients, 55 colorectal adenoma patients, and 51 healthy volunteers. The findings indicated that in all human serum samples, m^3^U, U_m_, m^6^A, m^5^U, U, A, C_m_, m^1^G, G_m_, m^2^G, and m^1^A were detected. As shown in Figure 3, the retention times of m^3^U, U_m_, m^6^A, m^5^U, U, A, C_m_, m^1^G, G_m_, m^2^G, and m^1^A were 2.01, 2.25, 3.65, 4.14, 4.96, 5.62, 6.49, 7.80, 8.58, 10.11, and 11.68 min, respectively. Notably, the chromatographic retention times of U_m_, m^6^A, m^5^U, U, A, C_m_, and m^1^A were consistent with their corresponding isotopically labeled internal standards. Moreover, m^3^U, m^2^G, G_m_, and m^1^G were confirmed by comparing their retention time with that of their standards (Supplementary Figure S1). In summary, the presence of these nucleosides in human serum was confirmed.

To determine whether there were any differences in the levels of these nucleosides between colorectal adenoma patients, CRC patients, and healthy controls, we quantified the levels of these nucleosides. First, the calibration curves were established, and the results showed that good linearity (*R* ^2^ > 0.999) was achieved (Table 1). The concentrations of nucleosides in serum were calculated according to the linear equations. For the quantification of m^3^U, [^13^C_5_] m^5^U was used as the internal standard. Also, for m^2^G, G_m_, and m^1^G, [D_6_] m^2,2^G was used as the internal standard. A comparison of the mean concentrations of A, m^1^A, m^6^A, U, m^3^U, m^5^U, U_m_, C_m_, m^2^G, m^1^G, and G_m_ in serum samples was shown in Supplementary Table S3. The levels of A, m^1^A, m^6^A, U, m^3^U, m^5^U, U_m_, C_m_, m^1^G, m^2^G, and G_m_ in the serum samples from healthy controls were within the range of 0.25–1.93, 24.93–127.80, 6.97–37.71, 2,170.83–9,368.89,6.48–62.79, 141.31–356.98, 11.45–64.80, 8.08–54.77, 14.02–38.11, 4.33–23.96, and 7.85–25.54 nM, respectively. The mean concentrations were 0.66, 64.70, 16.62, 4,803.45, 29.23, 225.57, 31.17, 25.69, 25.87, 14.04, and 14.80 nM, respectively. In the serum from patients with colorectal adenomas, the levels of A, m^1^A, m^6^A, U, m^3^U, m^5^U, U_m_, C_m_, m^1^G, m^2^G, and G_m_ were in the range of 0.18–1.18, 25.47–168.41, 6.36–51.35, 2039.52–7,855.11,4.36–76.20, 150.96–316.67, 6.76–55.78, 7.03–47.95, 12.49–43.36, 5.64–22.37, and 8.38–21.12 nM, respectively. The mean concentrations were 0.46, 77.32, 21.43, 4,634.80, 28.80, 210.41, 24.17, 26.89, 24.66, 13.85, and 13.18 nM, respectively. The concentrations of A, m^1^A, m^6^A, U, m^3^U, m^5^U, U_m_, C_m_, m^1^G, m^2^G, and G_m_ in the serum from patients with CRC were in the range of 0.08–0.82, 32.10–200.98, 8.01–69.87, 2,389.30–7,951.33, 24.93–119.88, 106.71–305.06, 5.02–18.67, 3.70–49.25, 13.93–49.65, 4.09–18.86, and 7.93–14.39 nM, respectively. The mean concentrations were 0.39, 105.85, 40.09, 4,377.36, 51.31, 206.68, 10.72, 24.75, 25.06, 9.67, and 11.09 nM, respectively.

**TABLE 1:** Linearity of A, m^1^A, m^6^A, U, m^3^U, m^5^U, U_m_, C_m_, m^2^G, m^1^G, and G_m_ in the LC-MS/MS analysis.

	Linear equation	*R* ^2^ value	Linear range (nM)
A	y = 0.139x − 0.0006	0.9997	0.1–5
m^1^A	y = 0.0082x − 0.0176	0.9991	1–250
m^6^A	y = 0.0183x − 0.0067	0.9991	1–100
m^5^U	y = 0.0173x − 0.0955	0.9993	1–500
m^3^U	y = 0.0392x − 0.0428	0.9991	1–250
U	y = 8.1622x + 0.2449	0.9999	100–5,000
U_m_	y = 0.0156x + 0.0077	0.9999	0.1–100
m^2^G	y = 0.001x + 0.0001	0.9999	1–100
m^1^G	y = 0.0095x + 0.001	0.9999	1–100
G_m_	y = 0.0046x − 0.0036	0.9993	1–100
C_m_	y = 0.0113x − 0.0076	0.9993	1–100

### 3.3 Evaluation of nucleosides as potential biomarkers of colorectal adenomas and CRC

**FIGURE 4:**
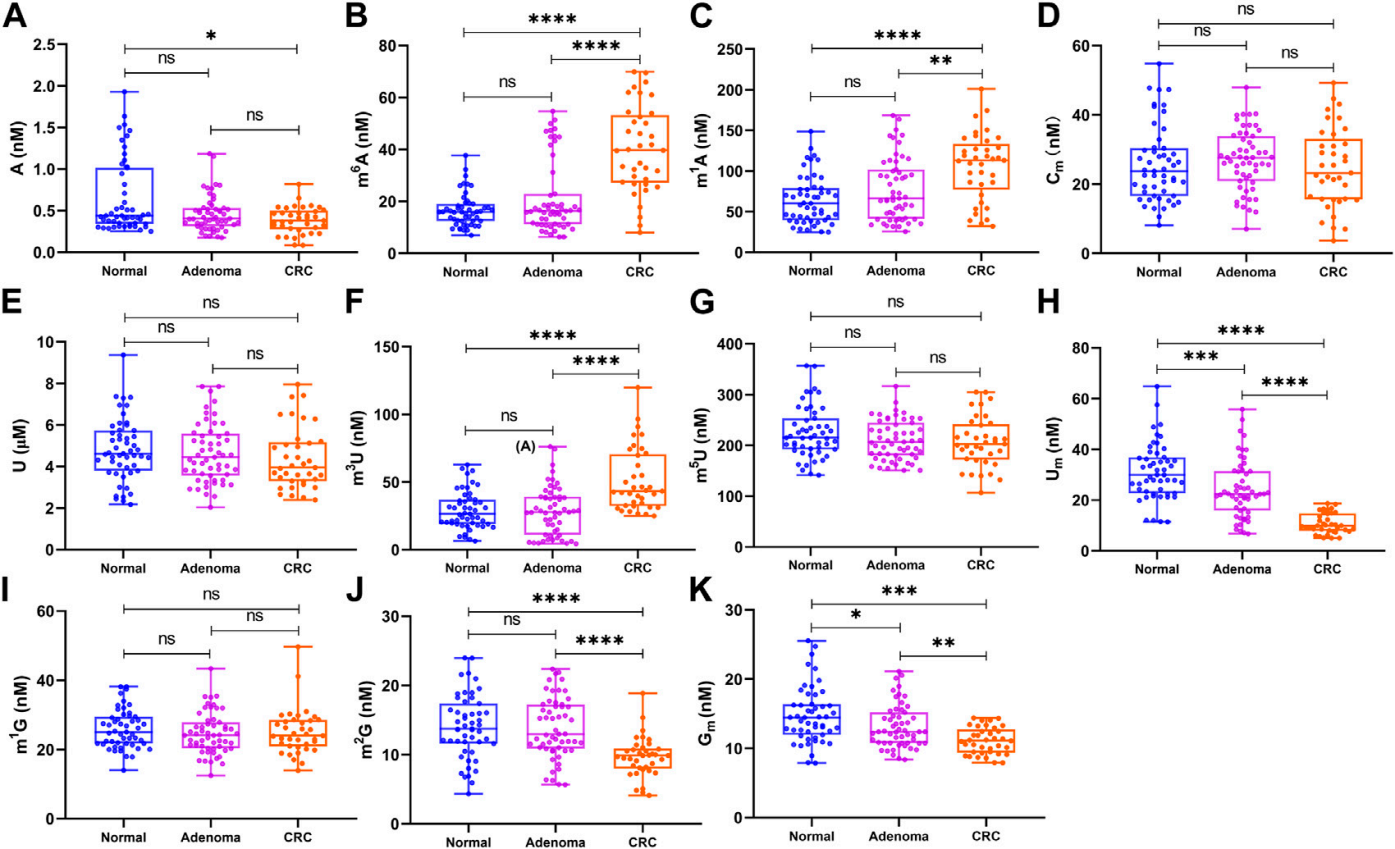
Concentration of **(A)** A, **(B)** m^6^A, **(C)** m^1^A, **(D)** C_m_, **(E)** U, **(F)** m^3^U, **(G)** m^5^U, **(H)** U_m_, **(I)** m^1^G, **(J)** m^2^G, and **(K)** G_m_ in the serum samples and statistical analysis. ****, *p* < 0.0001; ***, *p* < 0.001; **, *p* < 0.01; *, *p* < 0.05; ns, *p* > 0.05.

As shown in Figure 4, the levels of m^1^A, m^6^A, and m^3^U in the serum from patients with CRC were considerably higher than those of the healthy controls (*p* < 0.0001 for m^1^A, *p* < 0.0001 for m^6^A, and *p* < 0.0001 for m^3^U) and those of colorectal adenoma patients (*p* < 0.01 for m^1^A, *p* < 0.0001 for m^6^A, and *p* < 0.0001 for m^3^U). In contrast, the concentrations of U_m_, G_m_, and m^2^G in CRC patients were considerably lower than those of the healthy controls (*p* < 0.05 for A, *p* < 0.0001 for U_m_, *p* < 0.001 for G_m_, and *p* < 0.0001 for m^2^G) and those of colorectal adenoma patients (*p* < 0.0001 for U_m_, *p* < 0.01 for G_m_, and *p* < 0.0001 for m^2^G). Compared with healthy controls, the level of A was also decreased in the serum from CRC patients, whereas there was no difference between colorectal adenoma patients and CRC patients. Moreover, the levels of U_m_ and G_m_ were both decreased in colorectal adenoma patients, compared with the healthy controls. Interestingly, the concentrations of U_m_ and G_m_ decreased gradually from the healthy control group to colorectal adenoma patients to CRC patients. However, there were no differences in the levels of m^5^U, U, m^1^G, and C_m_ between these groups (*p* > 0.05).

**FIGURE 5:**
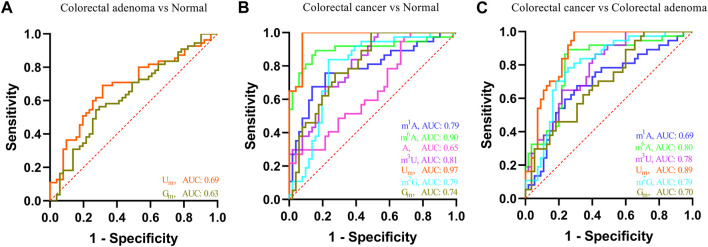
ROC analysis for these nucleosides in the serum. **(A)** Colorectal adenoma *vs.* normal control, **(B)** colorectal cancer *vs.* normal control, and **(C)** colorectal cancer *vs.* colorectal adenoma.

In addition, receiver operating characteristic (ROC) curve analysis was carried out to evaluate the capacity of these nucleosides to differentiate patients with colorectal adenomas and patients with CRC from the healthy controls. As shown in Figure 5A, for colorectal adenoma patients and normal controls, the area under the curve (AUC) of U_m_ and G_m_ were 0.69 and 0.63, respectively. For CRC patients and normal controls (Figure 5B), the AUC of m^1^A, m^6^A, A, m^3^U, U_m_, m^2^G, and G_m_ were 0.79, 0.90, 0.65, 0.81, 0.97, 0.79, and 0.74, respectively. For CRC patients and colorectal adenoma patients (Figure 5C), the AUC of m^1^A, m^6^A, m^3^U, U_m_, m^2^G, and G_m_ were 0.69, 0.80, 0.78, 0.89, 0.79, and 0.70, respectively. These results indicate that there is a close association between the levels of these nucleosides in the serum and the incidence of colorectal adenomas and CRC.

Both the morbidity and mortality rates of colorectal cancer are on the rise around the globe. Timely detection of specific biomarkers plays a vital role in early diagnosis, prevention, and monitoring during treatment. According to the findings of this study, an increase in m^6^A, m^1^A, and m^3^U in the serum and a decrease in A, U_m_, G_m_, and m^2^G in the serum, may have great potential as non-invasive biomarkers for the early detection of CRC. Notably, decreased levels of U_m_ and G_m_ may serve as non-invasive biomarkers for the early detection of colorectal adenomas.

## 4 Conclusion

In the present study, a targeted metabolomics method for the quantitative analysis of nucleosides in human serum samples by using LC-MS/MS was developed. We measured the concentration of A, m^1^A, m^6^A, U, m^3^U, m^5^U, U_m_, C_m_, m^1^G, m^2^G, and G_m_ in the serum samples from 51 healthy volunteers, 37 CRC patients, and 55 colorectal adenoma patients. CRC patients had significantly higher levels of m^6^A, m^1^A, and m^3^U than the colorectal adenomas patients and healthy volunteers. The levels of U_m_, G_m_, and m^2^G in CRC patients were significantly lower than those in the colorectal adenomas patients and healthy volunteers. Interestingly, there was a gradual decrease in U_m_ and G_m_ concentrations from healthy controls to colorectal adenoma patients to CRC patients. All these results suggested that the levels of m^6^A, m^1^A, m^3^U, A, U_m_, G_m_, and m^2^G in the serum were expected to be potential non-invasive biomarkers for the early detection of CRC, and the levels of U_m_ and G_m_ might be non-invasive biomarkers for the early detection of colorectal adenomas. In addition, these results implied that these nucleosides might play an important role in the development and progression of colorectal adenomas and CRC.

## Data Availability

The original contributions presented in the study are included in the article/Supplementary Material, further inquiries can be directed to the corresponding author.
