# Supplementary material for: Targeted metabolomics analysis of nucleosides and the identification of biomarkers for colorectal adenomas and colorectal cancer
Source: Front Mol Biosci. 2023 Jun 27;10:1163089. doi: 10.3389/fmolb.2023.1163089 (PMC10334214; doi:10.3389/fmolb.2023.1163089)
Supplement: Supplementary file 4 [file Table4.DOCX]

**Supporting Information**

**Targeted metabolomics analysis of nucleosides and identification of biomarkers for colorectal adenomas and colorectal cancer**

Weifang Zheng^1#*^, Mingwei Wang^2#^, Xiaoyin Chai^3^, Fuzhen Pan^1^, Meihui Xu^1^, Yingchen Wang^1^, Liuhao Lan^3^, Feiran Hu^1^, Zhe Zhang^3^, Zhu Chen^1^

^1^Lanxi Hospital of Traditional Chinese Medicine, Jinhua, China.

^2^College of Chemical Engineering, Zhejiang University of Technology, Hangzhou, China.

^3^Lanxi Red Cross Hospital, Jinhua, China.

^#^These authors contributed equally.

^*^Corresponding author:

Weifang Zheng, zhengweifang1972@163.com

**Table of Contents**

**Table S1.** The basic information of individuals recruited.

**Table S2.** The optimized MRM parameters applied to the analysis of nucleosides and their isotope labelled internal standards.

**Table S3.** The mean concentrations of A, m^1^A, m^6^A, U, m^3^U, m^5^U, U_m_, C_m_, m^2^G, m^1^G and G_m_ in serum from patients with colorectal cancer, patients with colorectal adenoma and healthy controls.

**Fig. S1.** Identification of m^3^U, G_m_, m^1^G and m^2^G in human serum. Representative MRM chromatograms of m^3^U, Gm, m^1^G, and m^2^G (A) in a serum sample, and (B) standard.

**Table S1.** The basic information of individuals recruited.

| **Group** | **Healthy control** | **Colorectal cancer** | **Colorectal adenoma** |
| --- | --- | --- | --- |
| Number of cases | 51 | 37 | 55 |
| Age (year) | 65.6± 2.4 | 66.0± 6.0 | 65.2± 7.7 |
| Gender  (Male/Female) | 37/16 | 23/16 | 37/20 |

**Table S2.** The optimized MRM parameters applied to the analysis of nucleosides and their isotope labelled internal standards.

| **Compound** | **MRM**  **ion transition (*m/z*)** | | **CE (V)** | **DP (V)** | **EP (V)** | **CXP (V)** |
| --- | --- | --- | --- | --- | --- | --- |
| m^1^A | 282.1→150.0 | 25 | | 55 | 10 | 10 |
| [D_3_]m^1^A | 285.1→153.0 | 25 | | 50 | 10 | 10 |
| m^6^A | 282.1→150.0 | 25 | | 55 | 10 | 10 |
| [D_3_]m^6^A | 285.1→153.0 | 25 | | 50 | 10 | 10 |
| m^3^U | 259.1→127.0 | 16 | | 45 | 7 | 10 |
| m^5^U | 259.1→127.0 | 16 | | 40 | 9 | 9 |
| [^13^C_5_]m^5^U | 264.1→127.0 | 20 | | 45 | 8 | 10 |
| U_m_ | 259.1→113.0 | 18 | | 45 | 6 | 16 |
| [D_3_]U_m_ | 262.1→113.0 | 12 | | 40 | 8 | 9 |
| m^1^G | 298.1→166.0 | 20 | | 55 | 7 | 14 |
| m^2^G | 298.1→166.0 | 22 | | 55 | 6 | 13 |
| G_m_ | 298.1→152.0 | 16 | | 50 | 7 | 12 |
| [D_6_]m^2,2^G | 318.1→186.0 | 30 | | 60 | 10 | 14 |
| C_m_ | 258.1→112.0 | 16 | | 45 | 10 | 8 |
| [D_3_]C_m_ | 261.1→112.0 | 18 | | 40 | 10 | 8 |

*CE* collision energy, *DP* declustering potential, *EP* entrance potential, *CXP* collision cell exit potential

**Table S3.** The mean concentrations of A, m^1^A, m^6^A, U, m^3^U, m^5^U, U_m_, C_m_, m^2^G, m^1^G and G_m_ in serum from patients with colorectal cancer, patients with colorectal adenoma and healthy controls.

|  | **healthy controls** | **colorectal adenoma** | **colorectal cancer** |
| --- | --- | --- | --- |
| A | 0.66 | 0.46 | 0.39 |
| m^1^A | 64.70 | 77.32 | 105.85 |
| m^6^A | 16.62 | 21.43 | 40.09 |
| m^5^U | 225.57 | 210.47 | 206.68 |
| m^3^U | 29.23 | 28.80 | 51.31 |
| U | 4803.45 | 4634.80 | 4377.36 |
| U_m_ | 31.17 | 24.17 | 10.72 |
| m^2^G | 14.04 | 13.85 | 9.67 |
| m^1^G | 25.87 | 24.66 | 25.06 |
| G_m_ | 14.80 | 13.18 | 11.09 |
| C_m_ | 25.69 | 26.89 | 24.75 |


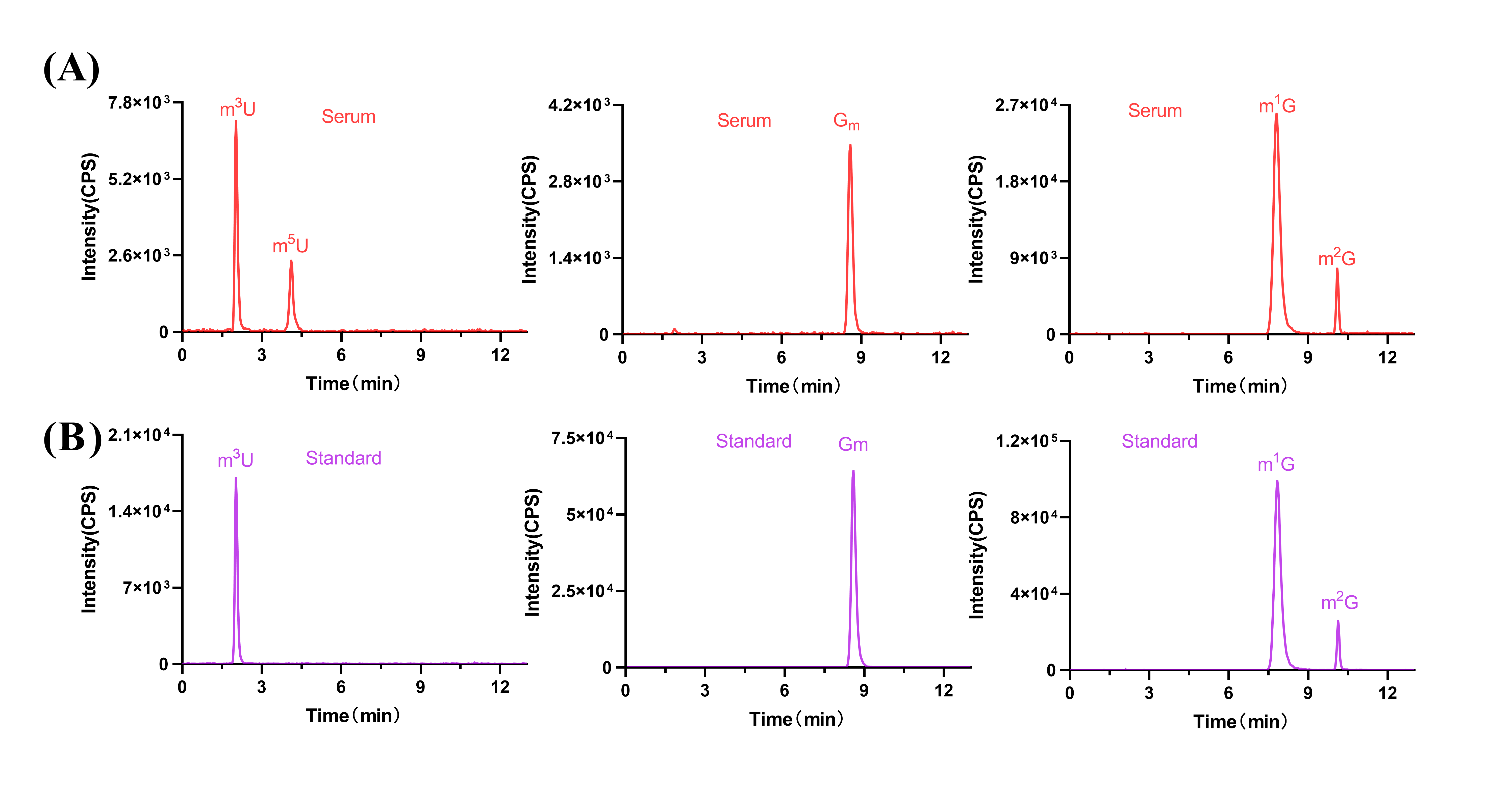
**Fig. S1.** Identification of m^3^U, G_m_, m^1^G and m^2^G in human serum. Representative MRM chromatograms of m^3^U, Gm, m^1^G, and m^2^G (A) in a serum sample, and (B) standard.
